# Combining Monte Carlo Simulation and Bayesian Networks Methods for Assessing Completion Time of Projects under Risk

**DOI:** 10.3390/ijerph16245024

**Published:** 2019-12-10

**Authors:** Ali Namazian, Siamak Haji Yakhchali, Vahidreza Yousefi, Jolanta Tamošaitienė

**Affiliations:** 1 Department of Industrial Engineering, College of Engineering, University of Tehran, Tehran 1417414418, Iran; yakhchali@ut.ac.ir; 2 Project Management, University of Tehran, Tehran 1417414418, Iran; vr.yousefi@ut.ac.ir; 3 Civil Engineering Faculty, Vilnius Gediminas Technical University, LT 2040 Vilnius, Lithuania; jolanta.tamosaitiene@vgtu.lt

**Keywords:** risk analysis, risk interactions, project completion time, Monte Carlo simulation, Bayesian networks

## Abstract

In this study, Monte Carlo simulation and Bayesian network methods are combined to present a structure for assessing the aggregated impact of risks on the completion time of a construction project. Construction projects often encounter different risks which affect and prevent their desired completion at the predicted time and budget. The probability of construction project success is increased in the case of controlling influential risks. On the other hand, interactions among risks lead to the increase of aggregated impact of risks. This fact requires paying attention to assessment and management of project aggregated risk before and during the implementation phase. The developed structure of this article considers the interactions among risks to provide an indicator for estimating the effects of risks, so that the shortage of extant models including the lack of attention to estimate the aggregated impact caused by risks and the intensifying impacts can be evaluated. Moreover, the introduced structure is implemented in an industrial case study in order to validate the model, cover the functional aspect of the problem, and explain the procedure of structure implementation in detail.

## 1. Introduction

Project-based organizations select and implement different projects to achieve their qualitative and quantitative goals. By assessing the real conditions of these projects, it can be claimed that organizations are rarely successful in on-time completion besides satisfying the financial and qualitative goals. One of the most important factors that leads to the incomplete completion of most projects is the lack of paying attention to the effects of risks in projects, so that the aggregated effect prevents desired completion of projects. Managing the existing risks has an important role in determining the success level of projects, especially construction projects. In other words, the interactions among risks lead to the increase of aggregated effect greater than the total effects of individual risks that is not much considered in proposed approaches of project risk assessment.

In practical cases, risks may affect each other due to extant interactions among them. Thus, an interaction network among risks must be identified in order to estimate the probabilities and effects of risks. For example, consider two risks: “Exchange rate changes“ and “Company financial problems” in a construction project. The occurrence of the first risk intensifies the probability and effect of the second risk. Therefore, the aggregated effect of these risks is greater than the sum of their individual effects. This aggregated effect is an influential factor in estimating project completion time. To estimate project completion time, the impacts of risks on the duration of activities must be identified. In order to assess the impact of risk on the duration of an activity, two approaches including risk-driven and activity-based approaches can be used. As proved by [1] a risk-driven approach is more effective than an activity-based approach when it comes to analyzing risks. Regarding this approach, we assess the impacts of risks directly instead of estimating distribution of activity durations based on the impacts of uncertain factors.

To the best of our knowledge, these practical cases have not been considered by researchers in the project risk assessment when it comes to estimating project completion time. Various techniques have been proposed for project risk assessment. In most of these techniques, the risks are analyzed separately, and the interactions among them have not been considered. One of the project risk assessment methods involving the possibility of modeling the interactions among risks is the Bayesian network approach. In previous studies in which Bayesian networks have been used to assess risks, only risk probability has been taken into account. Simulation methods such as Monte Carlo simulation are used to assess the effects of risks on functional goals of projects without considering the interactions among risks. On the other hand, the Bayesian network approach was developed to model the relationships between variables and can be used as an appropriate tool for considering the interactions among risks. In this study, by combining the methods of Monte Carlo simulation and Bayesian network, an approach is developed for assessing the aggregated effect of risks on a construction project completion time. By application of the Bayesian network logic, this study presents a new model of the project risk assessment in which the probabilities, consequences, and interactions among risks are simultaneously considered.

The problem can be described as follows. Suppose a set of activities of a project is available in which each activity has a predetermined duration. There is also a risk network which states potential risks, as well as the interactions among them. Each activity can be affected by one or more risks. As a result, the actual duration of each activity and the project completion time depend on the risk network structure. The research aim is to provide an approach for estimating the project completion time based on the project risk analysis and determining most effective risks on the timely completion of the construction projects.

The rest of this paper is organized as follows: The literature is reviewed in Section 2. In Section 3, the Bayesian belief network approach is introduced. In Section 4, the developed model for project risk assessment is presented. In Section 5, as an illustration, the developed model is performed in a real case study. Moreover, sensitivity analysis is discussed in this section and finally the paper is concluded in Section 6.

## 2. Literature Review

Construction is one of the most dangerous industries in the world [2]. The increasing complexity and dynamics of construction projects have plagued this industry with substantial risks and losses [3]. Therefore, the risk assessment in the construction projects, especially oil and gas refinery construction projects, is getting a matter leading from the importance and sensitivity of these projects duo to high investment and strong relationships with different internal and external suppliers. Then, the choice of proper risk measure impacts the portfolio in project-based organizations [4]. In construction projects, risks may dramatically impact on operation requiring an unexpected time for reconstruction resulting in primary cost and schedule delays that requires the need to design an approach for risk assessment.

Several techniques and models have been proposed for the project risk assessment. Multiple Criteria Decision Making (MCDM) is one of the approaches widely used to assess risks.

Aminbakhsh, Gunduz, and Sonmez [5] presented a framework for safety risk assessment by application of the Cost of Safety (COS) method and the Analytic Hierarchy Process (AHP) to prioritize safety risks of construction projects so that the practical objectives can be set without sacrificing safety. Zeng, An, and Smith [3] developed a risk assessment method to confront risks associated with the construction projects in the intricate situations. In their study, fuzzy reasoning and AHP approach were used to propose a risk assessment model in which fuzzy reasoning technique acts as a helpful tool for handling the uncertainties in the construction projects. Huang et al. [6] used a Delphi method and AHP approach to propose a risk analysis framework for Enterprise Resource Planning (ERP) projects. In the developed framework, a Delphi method is used to identify risks and AHP is used for assessment and then prioritization of the identified risks.

Kuo and Lu [7] applied a Fuzzy Multiple Criteria Decision Making (FMCDM) method to evaluate risks in a metropolitan construction project. In their research, in addition to using Consistent Fuzzy Preference Relations (CFPR) for measuring and investigating the impact of detected risks on the efficiency of project, the Fuzzy Multiple Attributes Direct Rating (FMADR) method was used to assess the probability of multi-risk occurrence. Zavadskas, Turskis, and Tamošaitiene [8] studied risk assessment for construction projects. For this purpose, they applied Technique for Order of Preference by Similarity to Ideal Solution (TOPSIS) grey and COmplex PRoportional ASsessment of alternatives with Grey relations (COPRAS-G) methods where the risk evaluation attributes were selected by regarding to the interests of the stakeholders and also the factors that can impress the efficiency of construction process.

Failure Mode and Effective Analysis (FMEA) is also used by some scholars to assess risks of projects. FMEA is a design tool used to systematically analyze postulated component failures and identify the resultant effects on system operations. Cheng and Lu [9] combined FMEA with fuzzy inference to propose a risk assessment method for pipe jacking construction projects. For identification and prioritization of technical risks, this model used a three-step framework to map the relationship among occurrence (O), severity (S), and detection (D) with the degree of criticality of risk events. Jamshidi et al. [10] introduced a unified framework based on combining fuzzy FMEA, and Grey Relational Analysis (GRA) to analyze risk in ERP projects by considering uncertainties. This framework proposed a systematic procedure for considering interdependence in the risk assessment process. Yang et al. [11] suggested a systematic assessment of risks in ERP projects via the use of FMEA technique. In the developed framework, a performance matrix was built compatible with the places of the three Risk Priority Number (RPN) indicators in the performance evaluation matrix.

As another approach for assessing risks, Fault Tree Analysis (FTA) has been used in which an undesired state of a system is analyzed using Boolean logic to combine a series of lower-level events. Gierczak [12] developed a mathematical model based on the FTA and fuzzy sets theory for the qualitative and quantitative risk assessment of the horizontal directional drilling projects in diverse sizes. In the presented model, fuzzy set theory was applied to reduce the uncertainty of the conventional FTA. Hyun et al. [13] proposed a risk management system applicable in tunnel boring machine tunneling. In their study, having categorized risks, a risk assessment approach was proposed by application of FTA and AHP in which the probabilities and impacts of risk events are taken into consideration.

Liang et al. [14] by using the Self-Organizing Map (SOM) and FTA techniques, developed an approach to analyze the third-party interference risk in long transmission pipeline projects. In the proposed approach, FTA and SOM were used to establish the risk evaluation index system and classify the multi-parameter risk pattern. They claimed that the methodology is able to detect existing relationships among risk-related variables and respective risk pattern in these projects. Zeng and Skibniewski [15] tried to model the relationships among ERP system components and particular risk events to propose an FTA-based risk assessment approach for ERP system implementation projects. Their approach can be used to quantify the adverse impact of risk factors on ERP component failures and subsequently to calculate the probability of the entire ERP system usage failure.

Mentioned approaches for assessing project risks, often analyze the risks without considering interaction relationships among them while in practical cases risks are correlated and sequentially the probability or impact of a risk must be estimated regarding to the occurrence or nonoccurrence of its parent risks. For this purpose, Monte Carlo simulation and Bayesian network (BN) methods are applicable. Monte Carlo simulation is a computational approach based on the repeated random sampling and statistical analysis to obtain numerical results. The underlying concept is to use randomness to solve problems that are impressed by uncertain parameters, many of which are difficult to obtain experimentally. Monte Carlo simulation performs risk analysis by building models of possible results by substituting a range of values for any uncertain factor. Some scholars have used this method to perform risk analysis. Sadeghi, Fayek, and Pedrycz [16] for dealing with the problem of handling both random and fuzzy uncertainties in a risk analysis process, proposed a Fuzzy Monte Carlo Simulation (FMCS) framework for risk assessment of construction projects. In the developed framework, a fuzzy cumulative distribution function was used to represent uncertainty in the risk assessment. Finally, for the purpose of verifying the feasibility of the FMCS framework, a simulation template was developed for estimating the cost of a highway overpass project.

In addition to simulation methods, the Bayesian network (BN) approach has been utilized to model the interaction relationships among risks. BN is a probabilistic graphical model that offers a compact presentation of the interactions in a stochastic system by visualizing system variables and their dependencies via a directed acyclic graph. Leu and Chang [17] proposed a model to construct a Bayesian network based safety risk-assessment model for steel construction projects. The Bayesian network model based on the fault tree transformation was authenticated by nine steel construction building projects in which particular incidents occurred at each steel construction site. They concluded that transformation from a fault tree to Bayesian network can construct a practical and precise safety risk-assessment model.

Hu et al. [18] studied causality analysis among risk factors and project outcomes for software development projects. For this purpose, they proposed a modeling framework based on the Bayesian network to deal with causality constraints in risk analysis. The developed framework can be used for discovering new causal relationships and validating existing relationships among risk factors and project outcomes. The authors of [19] developed a project portfolio risk assessment model based on the Bayesian network approach to indicate the causes of delays in construction projects.

Kim, Van Tuan, and Ogunlana [20] described how the Bayesian network can be applied to predict the probability of schedule delay of construction projects in a developing country. For this purpose, sixteen factors and eighteen cause–effect relationships between these factors were identified to present a Bayesian network model. Moreover, the developed model was validated through two practical case studies in Vietnam. According to the result of sensitivity analysis they stated that construction delay is sorely sensitive to some factors such as ‘shortage of materials’, ‘defective construction work’, and ‘slow site handover’. Chin et al. [21] modeled crucial risk factors in new product development project and their interrelationship into a Bayesian network to facilitate assessment of the risks in a new product development process. An approach was proposed to generate prior and conditional probabilities for the no-parent and multi-parent nodes in a Bayesian network. Finally, an industrial case study was used to illustrate the efficiency of the model. The mentioned literature review is summarized in Table 1.

In the developed risk assessment techniques that have used Bayesian Network logic, risk assessment has only been carried out based on the probabilities of risks and the consequences of risks on project activities have not been taken into account. Moreover, other project risk assessment techniques have shortage of not considering interactions among risks.

Despite the advantages of the proposed approaches, there are drawbacks. In simulation methods such as Monte Carlo simulation a distribution function must be defined for each effective risk. Owing to the lack of historical data, the determination of a distribution function is not a straightforward task. On the other hand, in methods in which Bayesian networks have been used to assess risk, only the probabilities of risks are considered and their impacts have not been taken into account. 

Given the real situation of projects in which risks are not independent and affect each other, there is a need to consider the network of project risks as representing how one risk can affect the probabilities and impacts of other risks. For project risk assessment, each risk should be evaluated based on the probabilities and impacts of other risks. In this study, a Bayesian network approach is used to calculate the probability part of project risk regarding the interaction structure of risks. The joint probability of a risk is determined by multiplying their conditional probabilities. The probability of each risk is calculated as the condition of occurrence or nonoccurrence of its affective risks. The Bayesian network method also has been used to consider the interaction structure between risks in calculating the probability of project portfolio risk. This study developed an approach to assess project schedule delay risk by considering the effects besides the interactions among risks and an equation is presented to evaluate the aggregated impact of risks for all scenarios of risk occurrence. Moreover, a project of gas refinery construction will be used as an industrial case study in order to explain and validate the presented model.

## 3. Bayesian Network Approach

Bayesian networks or causal networks are graphical representations of inferred knowledge for decision analysis in conditions of uncertainty [25]. The Bayesian network method is a modelling approach for presenting interactions between variables in a probabilistic system by visualizing system variables and their interactions [26]. The Bayesian network method is based on the Bayesian formula. It can be used to express the relationship between the prior probability and conditional probability of a given node and also to derive the incidence probability of a specific event related to that node [27].

For the *n* mutually restricted hypotheses (*j* = 1, 2,…, *n*) Bayes’ theorem is represented by the relationship:

(1) $$P\left(H_{j}|E\right)=\frac{P\left(E|H_{j}\right)\times P\left(H_{j}\right)}{\sum_{i=1}^{n}P\left(E|H_{i}\right)\times P\left(H_{i}\right)}$$

where $P\left(H_{j}|E\right)$ is the conditional probability for the hypothesis *H* (*j* = 1, 2,…, *n*), regarding the realized evidence (*E*); *P* (*Hj*) signifies the prior probability; $P\left(E|H_{j}\right)$ denotes conditional probability, assuming that $H_{j}$ is true, and the denominator denotes the total probability which is a constant value [28].

A Bayesian network is described by the qualitative and quantitative segments. In qualitative segment, the system variables and their dependences are represented through a directed acyclic graph (DAG) in which nodes and edges of the graph stand respectively for the system variables and their conditional dependences. In the quantitative segment, relationships among the nodes of the graph are stated by the conditional probability functions [26].

The starting nodes that have no inward arrow, are called the parent nodes. The child nodes are ones that have inward arrows which are connected to them. To calculate a specific probability, the states of each node and respective prior and conditional probabilities should be defined [29].

Considering the conditional dependencies of variables, Bayesian network represents the joint probability distribution *P* (*U*) of variables $U=\left\{A_{1},\ldots ,A_{n}\right\}$, as:

(2) $$P\left(U\right)=\overset{n}{\underset{i=1}{\prod }}P\left(A_{i}|Pa\left(A_{i}\right)\right)\;$$

where $Pa\left(A_{i}\right)$ is the parent set of variable $A_{i}$. Accordingly, the probability of $A_{i}$ is calculated as:

(3) $$P\left(A_{i}\right)=\underset{U\backslash A_{i}}{\sum }P\left(U\right)$$

where the summation is taken over all the variables except $A_{i}$.

**Figure 1:**
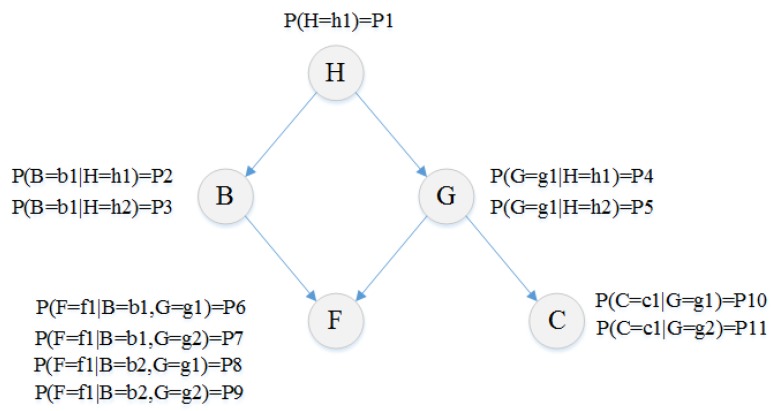
Bayesian network example.

Figure 1 is an illustration of a simple Bayesian network. This network consists of five binary variables in which the arrows connecting two variables, reflect the relations between them. In this example the arrow from B to F means that B has a direct impact on F and therefore the value of F depends on the value of B. Prior probability and conditional probabilities of variables are shown in this figure.

Regarding Figure 1, Bayesian network provides an appropriate structure for modeling dependencies between risks of the projects.

Furthermore, some advantages of BN includes the following [20]:

(1) Prior and conditional probabilities of variables in the Bayesian network can be developed using expert judgement instead of historical data; (2) modifications to the Bayesian network are isolated and thus variables can be added to or eliminated from a network without affecting the remainder of the network; (3) Due to the graphical display of Bayesian network relationships among the variables are very tangible; and (4) having constructed a Bayesian network, sensitivity analysis is capable of analyzing how much a particular node is influenced by other nodes.

## 4. Risk Assessment Model Structure

This section presents the developed simulation model for assessing project schedule delay risk. The model provides an indicator to evaluate the aggregated effect of project risks that could lead to an increase in the completion time of project. At first, the required parameters and equations are expressed and then the developed structure will be discussed.

### 4.1. Parameters

*R_p_*: Project risk level.
$R_{P}^{T}$: Time impact of project risk.
*R_k_*: Risk *k* of project.
$T_{t}$: The estimated project completion time.
*A_r_*: The set of risks affecting activity *r.*
*t_r_*: Start time of activity *r.*
*S_r_*: The set of successor activities of activity *r.*
*n:* The number of activities.
*d_r_*: The duration of activity *r* (by considering the effects of project risks).
$d_{r}^{0}$: The estimated duration of activity *r* (without considering the effects of project risks).
$R_{rk}^{\prime }$: The time impact of risk *k* on activity *r*.
$R_{rk}$: The aggregated time impact of risk *k* on activity.
$Pa\left(R_{k}\right)$: The set of parent risks of risk *k*.
$\propto_{Pa\left(R_{k}\right)}^{T}$: The increase in time impact of risk *k* in the event of its parent risk occurrence.
$val\left(R_{k}\right)$: Takes value 1 in the event of risk k and otherwise zero.
$val\left(Pa\left(R_{k}\right)\right)$: Takes value 1 in the event of parent risk of risk k and otherwise zero.

The project risk level is expressed in three levels including high (*h*), medium (*m*), and low (*l*) levels. With regard to Bayesian network computations, the probabilities related to three levels of project risk are as Equations (4) to (7).

(4) $$P\left(R_{P}=h\right)=\underset{R_{k}}{\sum }(\underset{k}{\prod }P\left(R_{k}|\mathrm{Pa}\left(R_{k}\right)\right)\cdot P\left(R_{P}=h|\mathrm{Pa}\left(R_{P}\right)\right))$$

(5) $$P\left(R_{P}=m\right)=\underset{R_{k}}{\sum }(\underset{k}{\prod }P\left(R_{k}|Pa\left(R_{k}\right)\right)\cdot P(R_{P}=m|Pa\left(R_{P}\right)))$$

(6) $$P\left(R_{P}=l\right)=\underset{R_{k}}{\sum }(\underset{k}{\prod }P\left(R_{k}|Pa\left(R_{k}\right)\right)\cdot P(R_{P}=l|Pa\left(R_{P}\right)))$$

The aggregated time impact of risk *k* on activity *r* and the duration of activity *r*, are as Equations (7) and (8), respectively.

(7) $$R_{rk}=val\left(R_{k}\right)\cdot R_{rk}^{\prime }\cdot \left(1+\underset{Pa\left(R_{k}\right)}{max}\left[val\left(Pa\left(R_{k}\right)\right)\cdot \propto_{Pa\left(R_{k}\right)}^{T}\right]\right)$$

(8) $$d_{r}=d_{r}^{0}\cdot \left(1+\underset{k\in A_{r}}{max}R_{rk}\right)$$

The aggregated impacts of risks represent the total impact for each risk including the primary impact and intensifying impact caused by their parent risks. Among impacts of parent risks, the largest one is considered. Given the duration required to perform each activity, the required duration for project completion will be calculated based on predecessor relationships between activities. The following modeling can be used to evaluate the completion time of the project:

$$mint_{n+1}$$

$$t_{j}-t_{i}\geq d_{i}\;\forall j\in S_{i}$$

(9) $$t_{i}\geq 0$$

where $t_{n+1}$ indicates the project completion time. Regarding Equations (4) to (7), the conditional expected values for project schedule delays are stated in Equations (10) to (12):

(10) $$E\left(R_{P}^{T}|R_{P}=h\right)=\underset{R_{k}}{\sum }(\underset{k}{\prod }P\left(R_{k}|Pa\left(R_{k}\right)\right)\cdot P(R_{P}=h|Pa\left(R_{P}\right))\cdot \left(t_{n+1}-T_{t}\right))$$

(11) $$E\left(R_{P}^{T}|R_{P}=m\right)=\underset{R_{k}}{\sum }(\underset{k}{\prod }P\left(R_{k}|Pa\left(R_{k}\right)\right)\cdot P(R_{P}=m|Pa\left(R_{P}\right))\cdot \left(t_{n+1}-T_{t}\right)$$

(12) $$E\left(R_{P}^{T}|R_{P}=l\right)=\underset{R_{k}}{\sum }(\underset{k}{\prod }P\left(R_{k}|Pa\left(R_{k}\right)\right)\cdot P\left(R_{P}=l|Pa\left(R_{P}\right)\right)\cdot \left(t_{n+1}-T_{t}\right))$$

Finally, according to Equation (13); the aggregated impact of the project schedule delay risk (e.g., the expected value of increase in project completion time) is based on Equation (14):

(13) $$E\left(x\right)=\underset{y}{\sum }E\left(x|Y=y\right)P\left(Y=y\right)$$

(14) $$E\left(R_{P}^{T}\right)=E\left(R_{P}^{T}|R_{P}=h\right)\cdot P\left(R_{P}=h\right)+E\left(R_{P}^{T}|R_{P}=m\right)\cdot P\left(R_{P}=m\right)+E\left(R_{P}^{T}|R_{P}=l\right)\cdot P\left(R_{P}=l\right)$$

This equation is obtained based on the conditional expected value formula in probability theory.

### 4.2. Steps of the Simulation Model

In this section, the proposed model is presented in which on-time completion of the construction project is considered as the project functional goal. The required steps for assessing the project schedule delay risk are as follows:

**Step 1:** Identify project activities: Since the effectiveness of risks on a project is realized by the effectiveness of risks on the project activities, it is necessary to specify these activities for assessing the effects of risks on the project. The time increase in activities affected by risks can cause a delay on project completion time. Consequently, at the first step, the project activities must be identified.

**Step 2:** Draw the project activity network: It is necessary to draw the project activity network in order to calculate the project completion time. This network indicates the precedence relationships between project activities.

**Step 3:** Identify risks: At this step, it is necessary to identify the risks raised at the project. There are different approaches to identify risks. One of those important approaches is risk breakdown structure which provides a structure to recognize risks systematically and promote the effectiveness and quality of the risk identification process.

**Step 4:** Identify interactions among risks: In risk management processes, the interaction among risks is often disregarded and just their probabilities and impacts are studied and assessed independently while the occurrence of a risk can intensify another risk. In other words, the probabilities and effects related to different risks should be determined according to the probabilities and impacts of other risks. Thus, it is necessary to design an interaction network among risks in order to estimate the probabilities and impacts of risks with higher accuracy.

**Step 5:** Identify the project activities affected by risks: As mentioned earlier, risks affect the project completion time through affecting its activity durations. As a result, for assessing the impacts of risks on project, the relationships between its activities and identified risks must be determined.

**Step 6:** Form the tables of prior and conditional probabilities: After drawing the project risk interaction network, including the identified risks with causal relations between them, it is necessary to determine the probabilities related to primary risks (risks without parents) and also secondary risks (risks with parents). For this purpose, the linguistic variables with equivalent numerical values can be used according to Table 2.

**Step 7.** Calculate the primary and intensifying impacts of risks: At this step, the main effects of each risk and their interaction effects are assessed. The main effects of each risk are determined as a value between 0 to 1 (0% to 100% effects) that indicates the effectiveness of that risk on the related activity time. Interaction effects indicate the increase of child risk effect in the case of its parent risk. Table 3 is used to assess the interaction effects between risks.

In Table 3, the value zero is related to the conditions when the occurrence of a risk affects the probability of another risk and has no effect on the effects of child risks.

**Step 8:** Cluster risks: This step is one of the main steps in the project simulation model. Risk clustering refers to the process of risk grouping at different levels according to the interaction relationships among them. These levels are as follows:

- First level: The risks that have no parents.
- K^th^ level: The risks that their parents are between the first level to (k − 1)^th^ level (for the second level to the last one).

**Step 9:** Produce random numbers (between 0 and 1) for all risks and determine the occurrence or nonoccurrence situation of risks according to prior and conditional probabilities: As was already mentioned, the first level risks are those that are not affected by other risks and act as effective risk on other risks. Secondary risks are ones that are affected by other risks (as child risk) and also can affect other risks (as parent risk). At this step, random numbers are produced for all these risks based on their prior and conditional probabilities. If the produced number for risks is less than the probability of occurring the related risk, the risk occurrence and otherwise nonoccurrence of the risk is assumed. This process is respectively done from the first level to the last one. At the last level, the status of the project risk will be determined in terms of high, medium, or low level of risk.

**Step 10:** Calculate the duration for implementing each activity and calculate the required time for completing the project: At this step, according to Equation (7), the aggregated effect of each risk on the related activity is calculated. This effect indicates the effects of each risk according to its main effect and the intensifying effects of its parent risks. By determining the required time to perform each activity (Equation (8)) and according to the project activity network, the calculation of the project completion time (e.g., the length of project critical path) will be possible (Equation (9)).

**Step 11:** Repeat steps 9 and 10 according to the number of pre-determined iterations: In order to realize a high level of reliability in the results, it is necessary to repeat the mentioned steps to a high degree in order to guarantee the reliability of the obtained results. Thus, the mentioned processes are done from the step of producing random numbers to the step of calculating the necessary time to complete the project with the pre-determined number of iterations.

**Step 12:** Calculate the value of project schedule delay risk: According to the proportion of each project risk level occurrence, the probability of the related level can be determined. In other words, if *N* (*R_P_* = *h*) shows the number of times in which the project risk is at a high level and *N_t_* shows the number of total repeats, the probability of the project risk at the high level is calculated according to Equation (15).

(15) $$P\left(R_{P}=h\right)=\frac{N\left(R_{P}=h\right)}{N_{t}}$$

Moreover, the average aggregated effects of risks can be calculated for each level. In other words, if $T_{t}\left(R_{P}=h\right)$ shows the total aggregated effects of risks (total delays in the project) for the number of repeats in which the level of the project risk is high, the average aggregated effects of risks (average delay in the project completion time) at this level is calculated by Equation (16).

(16) $$E\left(R_{P}^{T}|R_{P}=h\right)=\frac{T_{t}\left(R_{P}=h\right)}{N\left(R_{P}=h\right)}$$

The above calculations are done in the same way for other levels of risks. Finally, according to Equation (13), delay in the project is calculated as follows:

$$E\left(R_{P}^{T}\right)=E\left(R_{P}^{T}|R_{P}=h\right)\cdot P\left(R_{P}=h\right)+E\left(R_{P}^{T}|R_{P}=m\right)\cdot P\left(R_{P}=m\right)+E\left(R_{P}^{T}|R_{P}=l\right)\cdot P\left(R_{P}=l\right)$$

By replacing the equivalent values of the simulation model in the above equation, Equation (17) will be obtained:

(17) $$(R_{P}^{T})=\frac{N\left(R_{P}=h\right)}{N_{t}}\cdot \frac{T_{t}\left(R_{P}=h\right)}{N\left(R_{P}=h\right)}+\frac{N\left(R_{P}=m\right)}{N_{t}}\cdot \frac{T_{t}\left(R_{P}=m\right)}{N\left(R_{P}=m\right)}+\frac{N\left(R_{P}=l\right)}{N_{t}}\cdot \frac{T_{t}\left(R_{P}=l\right)}{N\left(R_{P}=l\right)}\;=\frac{T_{t}\left(R_{P}=h\right)+T_{t}\left(R_{P}=m\right)+T_{t}\left(R_{P}=l\right)}{N_{t}}=\frac{T_{t}}{N_{t}}$$

According to Equation (17), the project aggregated risk (project schedule delay risk) in the simulation model will be achieved through dividing the total aggregated effects of risks (total of delays) by the number of iterations.

**Figure 2:**
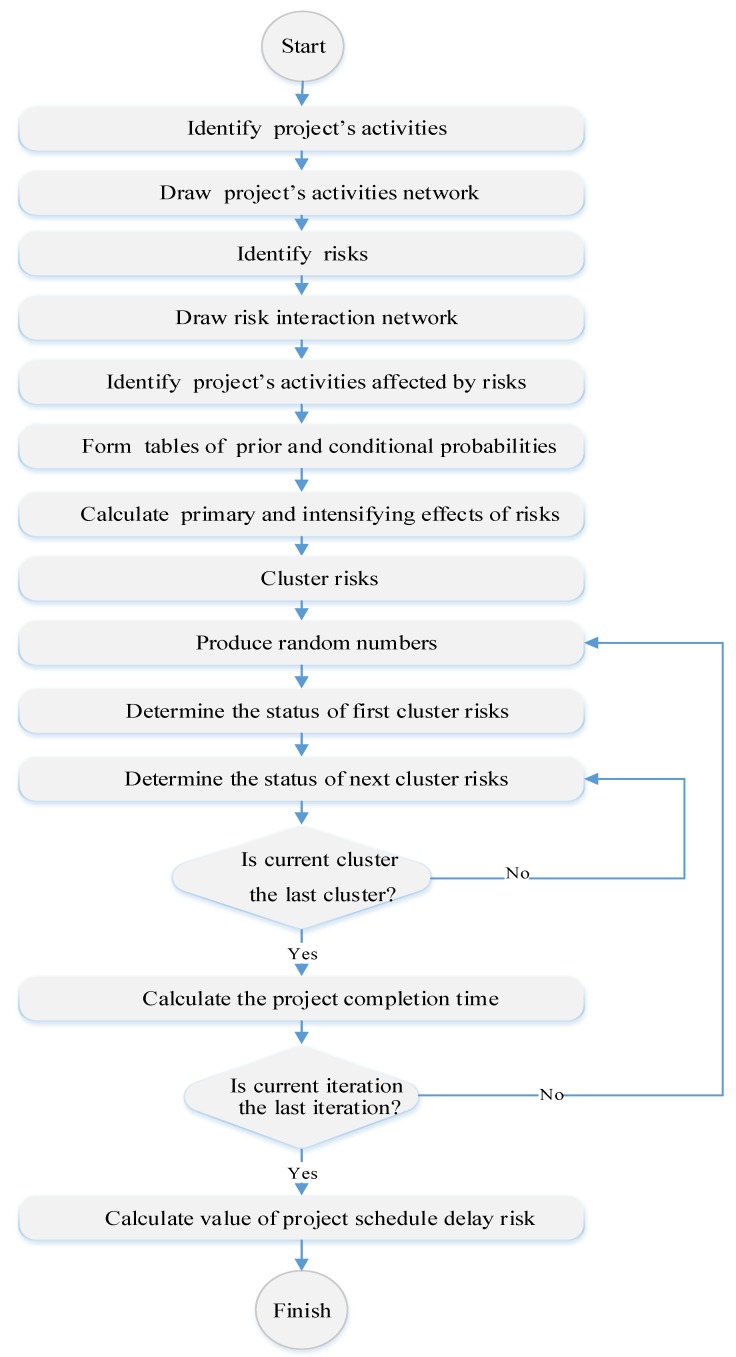
Project risk assessment flowchart.

The steps of simulation model described above, are summarized in Figure 2.

## 5. Model Implementation

As a practical illustration and validation of the developed model, in this section we present a real industrial case study. For this purpose, a project of gas refinery construction is considered to implement the model. In the following, the steps of the model are discussed.

**Step 1:** Identification of the project activities: Due to the large number of activities in a gas refinery construction project, in this problem work packages are considered as main activities. The project activities can be classified in three categories including design of technical drawings and codification of processes, materials and equipment procurement, and finally installation of the equipment in which the installation step consists of several activities such as piping, fix and rotary equipment installation, structure installation, painting, corrosion coating and insulation of pipes and equipment. The list of the activities with their estimated durations are shown in Table 4.

**Figure 3:**
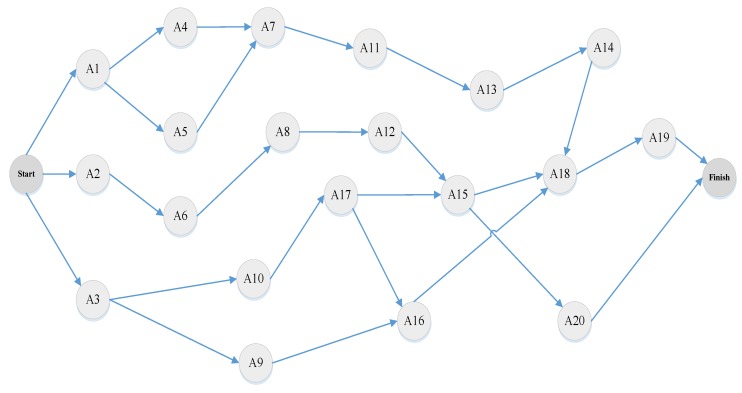
Project activity network.

**Step 2:** Drawing of the project activity network: The project activity network which indicates the precedence relationships between project activities is shown in Figure 3. This network is used to calculate the completion time of the project.

**Step 3:** Risk identification: The risks of the project identified by some methods such as risk breakdown structure (RBS), brainstorming and interview, are mentioned in Table 5. It should be noted that the selected project is done as an engineering, procurement, and construction (EPC) project, provided that the procurement phase has been outsourced to contracting companies.

**Figure 4:**
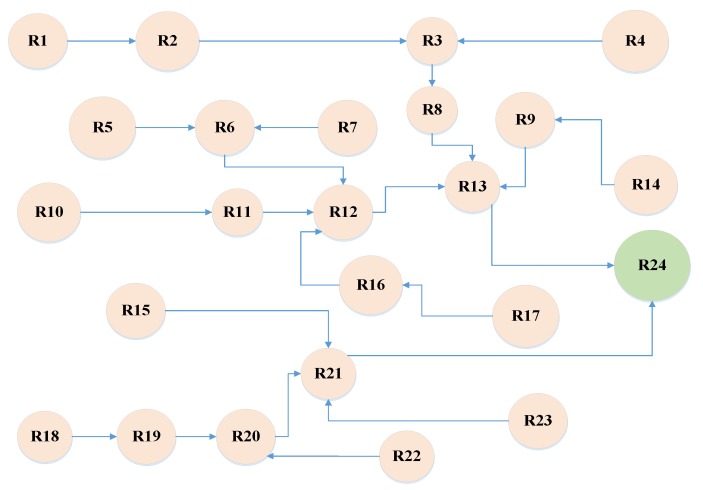
Risk interaction network.

**Step 4:** Identification of the interactions among risks: As mentioned earlier, the occurrence of a risk can intensify the probability and effect of another risk. For example, consider two risks; “Exchange rate changes “ and “Company financial problems”. In the case of the occurrence of the first risk, the probability and also effect of the second risk may be enhanced. Thus, it is necessary to design an interaction network among risks in order to estimate realistically the probabilities and effects of risks. The risk interaction network is shown in Figure 4.

**Step 5:** Identification of the project activities affected by risks: This step presents the relationships between activities and identified risks. For the mentioned project, the list of the activities with their affective risks are shown in Table 6.

**Figure 5:**
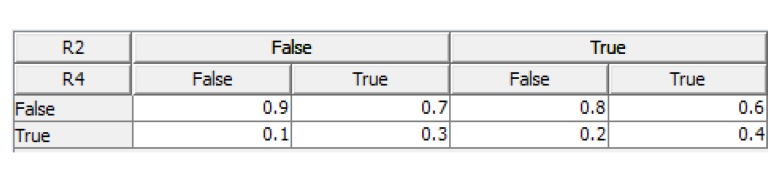
Conditional probabilities of risk R3.

**Step 6:** Formation of the tables of prior and conditional probabilities: In this section, the prior and conditional probabilities related to primary risks (risks without parents) and also secondary risks (risks with parents) are estimated regarding to the risk interaction network. For instance, Figure 5 shows the conditional probabilities of risk “Poor design“; (R3) regarding its parent risks: “Non-transparent and poorly defined objectives and scope of the project“; (R2) and “Weak communication between the executive team and design team“; (R4).

**Step 7.** Calculation of primary risks and intensifying effects of risks: Main effects of identified risks gathered from the project experts, are shown in Table 7. To simplify, it is assumed that the effect of each risk on the respective activities are similar.

According to Table 7, in the case of occurrence of the risk R1, durations of its affected activities will increase by 20%. Moreover, the interaction effects of risks are shown in Table 8.

**Step 8:** Risk clustering: Risk clustering refers to the process of risk grouping at different levels according to the interaction relationships among them in which no-parent risks appear at the first level and other risks appear at the levels that their parents belong to the previous ones. Project risk clustering is shown in Table 9.

**Step 9:** Determination of risk status regarding the generated random numbers: At this step, random numbers are produced based on prior and conditional probabilities of risks. If the produced number is less than the probability of occurring the respective risk, the risk occurrence and otherwise nonoccurrence of that risk is assumed. Random numbers generated in the first iteration of the simulation model and status of the respective risks are shown in Table 10. The occurrence and nonoccurrence of the risks indicate by numbers 1 and 0, respectively.

**Step 10:** Calculation of the duration for performing each project activity and the required time for completing the project: In this step, the project completion time is calculated regarding the aggregated effect of risks. In the first iteration of the simulation model, the project risk level is low and the total aggregated effects of risk is equal to 18 days which indicates 18-day delay in completion time of the project caused by risks.

**Step 11:** Repetition of steps 9 and 10 according to the pre-determined number of iterations: In this case, based on the similar studies, the number of iterations of 1000 is considered in order to guarantee the reliability of the obtained results.

**Step 12:** Calculation of the value of project schedule delay risk: Table 11 shows the obtained results of performing the simulation model by 1000 times (*Nt* = 1000). Moreover, the comparison of the values obtained from the simulation model with the exact values (the values obtained from Equations (4) to (12)), are listed in Table 12.

According to Table 12, the exact value of project schedule delay risk is 19.68 days and its value in simulation model is 19.93 days which verifies the high accuracy of the developed model. This value represents an increase of about 20 days compared to the anticipated project completion time.

### Sensitivity Analysis

Sensitivity analysis for the identified risks is performed to prioritize the risks in terms of their effects on the project aggregated risk. For this purpose, it is necessary to calculate the values of $E\left(R_{P}^{T}|R_{P}=h,R_{i}=0\right)$ and $E(R_{P}^{T}|R_{P}=h,{\;R}_{i}=1)$ for each risk. The wider range of changes for each risk factor indicates the higher priority of that factor. The range of changes for the first ten risks with the high priority are shown in Table 13.

According to Table 13, risks of “shortage of resources”, “the problems of the company in the project finance”, and “delay in technical drawings notification” are the most important risks affecting the project completion time that requires the adoption of some measures for preventing their occurrence. In other words, the risk of increasing the project completion time has the maximum sensitivity to the increase of times due to the shortage of resources, the financial problems of the company and delay in technical drawings notification, and is affected mostly by these factors more than other risk factors.

## 6. Conclusions

Projects are exposed by different risks during their implementations and often affect the functional goals of projects like the time or cost of the projects. Time and cost impacts of uncertain events have an important role in determining the success level of the projects. The adoption of some policies to manage these risks can be effective in eliminating or reducing their effects. In traditional approaches of risk management, the assessment of different risks is done independently and the interaction among them is not considered while the occurrence of some risks can increase the effect or probability of other risks. Some simulation methods have been proposed to provide an estimation of project completion time in which the activity-based approaches are used to assess the effects of risks regarding to distribution functions of activity durations. In practical cases, owing to the lack of historical data, the determination of a distribution function is not a straightforward task. On the other hand, in methods in which Bayesian networks have been used to assess risk, only the probabilities of risks are considered and their impacts have not been taken into account.

This article presented a structure combining Monte Carlo simulation and Bayesian Networks methods in order to assess the effects of risks, so that, the interactions among risks were considered. Unlike other methods of project completion time estimation based on the project risk assessment, this study provided an integrated 12-step approach in which a risk network mapped on the project activity network in order to estimate the effects of risks more accurately and obtain a more realistic estimate of time, when the project is completed.

The developed model can be used to assess the success level of the construction projects. The lower value of project risk level, the more achievements of the project goals will be for that level of uncertainty. Finally, this approach was implemented in an industrial case study in order to estimate the aggregated effect of risks on the project completion time. Comparing the results from the developed framework with the exact ones, verified the high accuracy of the model. Also, according to the performed sensitivity analysis, the prioritization of risks was determined in terms of the sensitivity of the project completion time to the occurrence of the risks. Also results stated that shortage of resources, the financial problems of the company and delay in technical drawings notification are the main causes of delay in an oil and gas refinery construction project.

The research results showed that integrated risk assessment regarding the activity and risk network of a project provides more accurate estimations of parameters affecting the project functional objectives and can be used by project managers to identify the influential risks and consequently enhance the probability of the project success.

Since the activity network of every project is influenced by uncertainties related to risk factors, the developed approach can be used to estimate project completion time of other projects. For this purpose, the risk factors and their interactions should be identified. By establishing the structure of how activities are affected by the risk factors, the schedule of activities and the project completion time can be estimated through modeling the project uncertainties.

However, this study had several limitations. Authors used 5-point scales for assessing the impacts and consequences of risks. It is better to generate risk parameters by using the stochastic programming models. Also, the developed model is applied at project level and can be used for analyzing uncertainties at project portfolio level. As a result, strategic decisions related to project portfolio risk assessment and project selection problem can be made by application of proposed approach and modeling the interactions among risks of different project.

As future researches, the presented approach of this study can be extended by; 1. Presenting a stochastic programming model to assess the aggregated risk of the project. 2. Developing a mathematical programming model to select projects with the minimum aggregated risk created from the selected projects. 3. Presenting an approach for integrated risk management in which different types of risks with extant effects and interactions are studied and analyzed.

## Figures and Tables

**Table 1:** Summary of literature review.

Paper	Risk Assessment Method							Project Type						
	MCDM	Fuzzy Set	ID	FMEA	FTA	Monte Carlo	BNs	Construction	ERP	IT	Drilling	Tunneling	Pipeline	Development
[3]	✓	✓						✓						
[5]	✓							✓						
[22]		✓	✓					✓						
[6]	✓								✓					
[7]	✓	✓						✓						
[23]	✓	✓								✓				
[8]	✓							✓						
[9]				✓				✓						
[10]	✓	✓		✓					✓					
[11]				✓					✓					
[12]		✓			✓						✓			
[13]	✓				✓							✓		
[14]					✓								✓	
[15]					✓				✓					
[24]						✓								✓
[16]		✓				✓		✓						
[17]							✓	✓						
[18]							✓							✓
[20]							✓	✓						
[21]							✓							✓

**Table 2:** Scales of prior and conditional probabilities.

Annual Frequency		Probability	
Descriptor	Definition	Descriptor	Value
Frequent	Up to once in one month or more	Very high	0.9
Likely	Once in one month up to once in six months	High	0.7
Possible	Once in six months up to once in twelve months	Medium	0.5
Unlikely	Once in twelve months up to once in twenty-four months	Low	0.3
Rare	Once in twenty-four months or less	Very low	0.1

**Table 3:** Interaction effect assessment of the parent risk on the child risk.

$\propto_{Pa\left(R_{k}\right)}^{T}$	0	0.2	0.4	0.6	0.8	1
Descriptor	Insignificant time increase	Very low (20% time increase)	Low (40% time increase)	Moderate (60% time increase)	High (80% time increase)	Very high (100% time increase)

**Table 4:** Project activities.

Parameter	Activity	Duration (Day)	Parameter	Activity	Duration (Day)
A1	Designing the technical drawings of pipes	30	A11	Procurement the pipes’ insulation	25
A2	Designing the technical drawings of fix and rotary equipment	75	A12	Procurement the equipment’ insulation	25
A3	Structure designing	60	A13	Above-ground and under-ground welding	80
A4	Pipes’ painting process codification	25	A14	Piping	110
A5	Pipes’ hot and cold insulation process codification	25	A15	Fix and rotary equipment installation	60
A6	Equipment’ hot and cold insulation process codification	30	A16	Air-cooling machine installation	50
A7	Pipes procurement	45	A17	Structure installation	40
A8	Fix and rotary equipment procurement	60	A18	Corrosion coating	30
A9	Air-cooling machine procurement	50	A19	Pipes’ hot and cold insulation	60
A10	Structure procurement	45	A20	Equipment’ hot and cold insulation	60

**Table 5:** Identified project risks.

Parameter	Risk	Parameter	Risk
R1	Incorrect understanding of project or employer expectations	R13	Delay in implementation of activities
R2	Non-transparent and poorly defined objectives and scope of the project	R14	Prolongation of the contract issues
R3	Poor design	R15	Problems in setting up the purchased and installed equipment
R4	Weak communication between the executive team and design team	R16	Incompetency of contractors
R5	Lack of incentive systems	R17	Drawback in the structure of tenders and selection of contractors
R6	Decline in labor productivity	R18	Employer budget deficit
R7	Drawback in educational systems	R19	Delay in payment by employer
R8	Delay in Technical Drawings Notification	R20	Company financial problems
R9	Increase in the time and financial claims by contractors	R21	Shortage of resources
R10	The unavailability of key labors at critical times of project	R22	Exchange rate changes
R11	The allocation of unskilled labor	R23	Late equipment delivery
R12	Low quality of implementation	R24	Delay in completion time of project

**Table 6:** Affective risks on project activities.

Activity	Affective Risks	Activity	Affective Risks
A1	R1 R2 R3 R4	A11	R14 R18 R19 R20
A2	R1 R2 R3 R4	A12	R14 R18 R19 R20
A3	R1 R2 R3 R4	A13	R10 R11 R21
A4	R11 R12	A14	R5 R6 R7 R10 R13 R15
A5	R11 R12	A15	R5 R6 R7 R11 R12 R21
A6	R11 R12	A16	R13
A7	R8 R9 R14 R16	A17	R5 R6 R7 R10
A8	R17 R18 R19 R20 R22	A18	R7 R11
A9	R14 R18 R19 R20	A19	R7 R11 R21
A10	R8 R9 R14 R16	A20	R7 R11 R21

**Table 7:** Main effect of risks.

Risk	Time Impact	Risk	Time Impact	Risk	Time Impact
R1	0.2	R9	0.2	R17	0.4
R2	0.4	R10	0.05	R18	0.1
R3	0.2	R11	0.1	R19	0.05
R4	0.4	R12	0.4	R20	0.2
R5	0.2	R13	0.8	R21	0.4
R6	0.2	R14	0.1	R22	0.4
R7	0.2	R15	0.4	R23	0.2
R8	0.1	R16	0.2		

**Table 8:** Interaction effects of risks.

Affective Risk	Affective Risk	Effect Intensity	Affected Risk	Affective Risk	Effect Intensity
R1	R2	0.2	R11	R12	0.2
R2	R3	0.2	R12	R13	0.2
R4	R3	0.2	R17	R16	0.2
R3	R8	0.4	R16	R12	0.4
R5	R6	0.4	R18	R19	0.2
R7	R6	0.4	R19	R20	0.2
R6	R12	0.4	R22	R20	0.2
R8	R13	0.6	R15	R21	0.2
R14	R9	0.2	R20	R21	0.4
R9	R13	0.4	R23	R21	0.6
R10	R11	0.2			

**Table 9:** Project risk clustering.

Level 1	Level 2	Level 3	Level 4	Level 5	Level 6
R1	R2	R3	R8	R13	R24
R4	R6	R12	R21		
R5	R9	R20			
R7	R11				
R10	R16				
R14	R19				
R15					
R17					
R18					
R22					
R23					

**Table 10:** Random numbers and status of risks in the first iteration.

Risk	R1	R2	R3	R4	R5	R6	R7	R8
Random number	0.376	0.645	0.856	0.156	0.982	0.461	0.369	0.19
Status of risk	0	0	0	0	1	1	1	0
Risk	R9	R10	R11	R12	R13	R14	R15	R16
Random number	0.669	0.882	0.561	0.033	0.265	0.617	0.29	0.252
Status of risk	0	1	1	0	0	1	0	0
Risk	R17	R18	R19	R20	R21	R22	R23	R24
Random number	0.583	0.385	0.226	0.59	0.121	0.482	0.428	0.191
Status of risk	0	1	1	1	0	0	0	0

**Table 11:** Simulation model results.

Parameter	Value
$N\;\left(R_{P}=m\right)$	383
$N\;\left(R_{P}=h\right)$	186
$N\;\left(R_{P}=l\right)$	431
$T_{t}\;\left(R_{P}=h\right)$	7645
$T_{t}\;\left(R_{P}=m\right)$	3765
$T_{t}\;\left(R_{P}=l\right)$	8522

**Table 12:** Simulation values versus exact values.

Parameter	Simulation Model Value	Exact Value
$P\;\left(R_{P}=h\right)$	0.383	0.3842
$P\;\left(R_{P}=m\right)$	0.186	0.1842
$P\;\left(R_{P}=l\right)$	0.431	0.4316
$E\;\left(R_{P}^{T}|R_{P}=h\right)$	19.96	20.08
$E\;\left(R_{P}^{T}|R_{P}=m\right)$	20.24	19.88
$E\;\left(R_{P}^{T}|R_{P}=l\right)$	19.77	19.24

**Table 13:** Sensitivity analysis of risks.

Risk	$E\left(R_{P}^{T}|R_{P}=h,R_{i}=0\right)$	$E(R_{P}^{T}|R_{P}=h,\;R_{i}=1)$	Range of Changes
R21	19.772	20.116	0.344
R20	19.804	19.908	0.104
R8	19.800	19.884	0.084
R22	19.804	19.88	0.076
R9	19.816	19.86	0.044
R23	19.828	19.852	0.024
R6	19.824	19.844	0.020
R15	19.824	19.832	0.008
R3	19.832	19.836	0.004
R7	19.832	19.836	0.004
